# Roles and Challenges for Village Doctors in COVID-19 Pandemic Prevention and Control in Rural Beijing, China: A Qualitative Study

**DOI:** 10.3389/fpubh.2022.888374

**Published:** 2022-06-29

**Authors:** Jin Li, Ning Zhao, Haiyan Zhang, Hui Yang, Jia Yang

**Affiliations:** ^1^School of Public Health, Capital Medical University, Beijing, China; ^2^Department of Health Education, Beijing Huairou Hospital of University of Chinese Academy of Sciences, Beijing, China; ^3^Beijing Key Laboratory for Pediatric Diseases of Otolaryngology Head and Neck Surgery, National Center for Children's Health, Beijing Children's Hospital, Capital Medical University, Beijing, China

**Keywords:** rural area, village doctor, COVID-19, qualitative study, prevention and control of pandemics

## Abstract

**Objectives:**

Rural areas in China are more vulnerable to COVID-19 pandemic than urban areas, due to their far fewer health care resources. Village doctors, as rural grassroots health workers in China, have been actively engaged in the pandemic prevention and control. This study aims to describe the roles of village doctors in rural China, and the challenges they have faced during the prevention and control of the COVID-19 pandemic.

**Setting:**

This study was conducted in three towns in Huairou District, Beijing, China.

**Design:**

We carried out semi-structured interviews with 75 key informants. All the interviews were audio-recorded and transcribed verbatim. We employed thematic analysis to define themes and sub-themes from the qualitative data.

**Results:**

We reported four themes. First, the village doctor guided the village committee to carry out decontamination, monitored home-isolated residents, and disseminated knowledge on prevention of the COVID-19 pandemic during the rural pandemic prevention and control. Second, they took pandemic prevention measures in village clinics, distributed pandemic prevention materials, and undertook pre-screening triage. Third, village doctors provided basic medical care, including treatment of common diseases as well as the purchase and delivery of medicines to villagers. Fourth, village doctors faced difficulties and challenges, such as inadequate medical skills, aging staff structure, and lack of pandemic prevention materials.

**Conclusions:**

Despite many difficulties and challenges, village doctors have actively participated in rural pandemic prevention and control, and made outstanding contributions to curbing spread of COVID-19 pandemic in rural areas. Village doctors provide basic health care while participating in various non-medical tasks.

## Introduction

Since the first case of coronavirus disease 2019 (COVID-19) was detected in Wuhan, Hubei Province in December 2019, COVID-19 had spread rapidly worldwide (1), having a profound impact on daily lives. Many provinces in China have consecutively reported confirmed cases. In order to control the fast spread of COVID-19, China took the strictest, and most thorough prevention and control measures (2, 3). The Chinese government adopted such “non-medical” measures, as closing community, prohibiting social activities, and scanning “health codes”, which had proven to be significantly effective (4). In preventing and controlling COVID-19, rural areas cannot be ignored, despite its low population density, relatively fixed places for villagers' activities and low population mobility (5). One reason is that compared with urban areas, rural public health emergency system is much weaker, definitely increasing the risk of infection for rural residents (6). Additionally, rural populations don't have equal medical resources as those in urban areas making it more difficult to quickly detect pandemics and treat infected patients (7).

In late January 2021, there was a localized pandemic in Hebei Province, with more than 85% of infected patients coming from rural areas (8). Different from the early 2020 this time the pandemic broke out and spread widely in rural areas. As the “gatekeeper” of farmers' health at bottom of the rural three-tier medical service network, village doctors function as an important component of the rural pandemic prevention and control team (9). Village doctors are required to provide basic medical and public health services to rural residents including health education, assistance in the implementation of infectious disease reporting and other prevention and control measures, and responding to public health emergencies. The State Council also issued a series of policies during the pandemic that specified the responsibilities of village doctors in COVID-19 prevention and control. Those responsibilities include monitoring the health status of residents in home isolation, assisting in tracking close contacts and conducting epidemiological investigations, as well as improving health management of priority populations with chronic diseases (10, 11).

Village doctors in China, on the other hand, are facing difficulties and challenges. Currently, part of village doctors are transformed from “barefoot doctors” (12). Mostly overage, less educated, less qualified and incompetent in information technology. Those features affect the quality, efficiency and accessibility of basic public health services (13–16). Therefore, it is worth studying what village doctors do, what roles they play, and what new challenges they are facing during the COVID-19 pandemic.

Previous studies have researched the roles and challenges of medical workers in the prevention and control of COVID-19 pandemic. Scholars from China, UK, Ireland, Singapore and other countries have studied the barriers and coping strategies of primary health workers in COVID-19 pandemic control (17–19). Several studies have found that medical workers face high levels of work stress and psychological pressure and lack of social support during the COVID-19 pandemic (20–23). Previous researches have researched the impact of the COVID-19 pandemic on special populations such as pregnant women, newborns, patients with chronic diseases, and school students (24–27).There is also research on how pandemic prevention and control is carried out in rural China and the challenges faced in rural areas (28). Hsu et al. and Putzer et al. argued that rural infrastructure and health resources were inadequate to respond to public health emergencies, and that vast majority of village doctors did not have confidence in diagnosing or treating public health emergency cases (29, 30). However, there is no literature on the participation of health workers in China in pandemic prevention and control in rural areas and the difficulties they encountered in rural settings.

According to the National Bureau of Statistics, by the end of 2020, China's rural population has reached 510 million, accounting for 36.1% of the total population. The official media reported that more than 4 million primary health workers in China have actively participated in the prevention and control of the pandemic, including 1.44 million village doctors, who play an important role in community prevention, control and screening personnel (31, 32).

Beijing has a population pattern of “big city, small rural area”. By the end of 2019, the resident population of Beijing was 21.536 million, including 2.886 million in rural areas. In the prevention and control of the COVID-19 pandemic, the prevention and control work in the capital city is vital, and making good efforts in its rural areas also can lay important foundation for the social stability in the capital city. Rural areas are the weak point of the health care system in Beijing, with relatively insufficient medical facilities and health manpower, and low awareness of disease prevention among rural residents. Remote rural areas have complex terrain, scattered dwellings and shortage of village doctors. Some mixed urban and rural areas are densely populated, highly mobile, with complex environments and insufficient quality medical resources. Hence there was greater pressure to prevent and control the COVID-19 pandemic in rural Beijing. Beijing has issued a series of documents requiring primary medical institutions to strengthen the monitoring and reporting of pandemics and to strictly manage fever and suspicious cases, as well as to strengthen rural pandemic prevention and control education and health management (33, 34).

As the first defense line of pandemic prevention and control in rural areas, village clinics and village doctors have made necessary contributions to the COVID-19 pandemic (35, 36). Hence, it is reasonable to have a comprehensive understanding of the roles of village doctors and challenges they may face in the prevention and control of COVID-19 pandemic, and offer suggestions for improving the rural public health emergency system.

## Materials and Methods

### Study Design

This study adopted a qualitative research method, using semi-structured interviews to collect respondents' views and experiences. Given the limited research related to the role of village doctors in rural pandemic prevention and control, we chose a thematic framework analysis approach to explore and analyze these issues (37, 38), because thematic framework analysis is particularly well suited for identifying policy- and practice-relevant findings (39). We designed a semi-structured interview based on relevant policies and several rounds of expert consultation. Specific questions were developed for different interviewees to understand the role of and problems from village doctors in pandemic prevention and control. We selected a village doctor, a village officer, a villager, a town-level community health center manager, and a town government manager for pre-interviews before the formal interviews. Major topics covered responsibilities of village doctors during the pandemic, changes in the number of patients who visited village clinics during the pandemic, the way how village doctors provided medical services during the pandemic, and difficulties encountered in providing medical care (see Supplementary Material).

### Study Area and Recruitment

This study was carried out in three towns in Huairou District, Beijing. Huairou District is a distant suburban district located in the northeast of Beijing, with a total area of 2,122.8 square kilometers, of which the mountainous area accounts for 89%. There are 14 towns and 284 administrative villages in the region, and a resident rural population of 121,000. Huairou is the region with low incidence of COVID-19 in Beijing. Until March 1, 2021, there were only 8 confirmed cases, ranking Huairou the last fourth in terms of case numbers. Based on the three aspects of geographic location, accessibility of health services, and the number of infected cases, three towns, X, Y and Z, were selected in Huairou District. X town belongs to shallow mountainous terrain with no COVID-19 cases reported. There are 21 administrative villages, with a permanent population of 15,754, and 21 village doctors aged 59.9 years on average of whom 4 are capable of doing pharyngeal swab sampling. Y town belongs to deep mountainous terrain with no COVID-19 cases reported. There are 24 administrative villages with a permanent population of 3,560, and 20 village doctors aged 64.7 years on average. All village doctors in Y town are not capable of doing pharyngeal swab sampling or epidemiological investigation. Z town belongs to plain terrain with reported COVID-19 cases. There are 18 administrative villages with a permanent population of 18,657, and 16 village doctors aged 64.2 years on average, all of whom cannot do pharyngeal swab sampling or epidemiological investigation. Interview sites were selected considering the village geography, size, population and distribution of village clinics. Five villages were selected in each town (2 large villages, 2 medium villages and 1 small village). Two towns government managers and two town-level health center managers were selected for each town. In each village, two villagers, a village officer and a village doctor were selected (6 village doctors in 3 villages in X town). All participants agreed to be interviewed after they understood the purpose of the study.

### Data Collection

The interview data were collected from March 2021 to July 2021, when Beijing has achieved the normalization of pandemic prevention and control, and the administrative village in Huairou District has also finished its closed management. All interviews were conducted face-to-face by interviewers who had been trained for conducting qualitative research. We trained a total of 12 interviewers. With the help of the district health administration, we also conducted semi-structured interviews with the community health center managers and government managers in each town. Before interviewing, we first explained the purpose of the study, voluntary participation, and principle of confidentiality to the interviewees and obtained their written informed consent. Afterwards, the interviews were noted and audio-recorded throughout the process. Interviews lasted 40–80 min.

### Data Analysis

The audio records of the interviews were transcribed verbatim and collated within 48 h. We developed a seven-stage analysis framework: (a) familiarizing with original materials; (b) identifying important themes or keywords; (c) listing original themes catalog or analytical framework; (d) coding original materials based on the theme catalog; (e) categorizing data based on themes or sub-themes; (f) summarizing or synthesizing data; (g) explaining data. Two researchers completed the previous five steps independently, and then invited several experts to compare codes, discuss existent disagreements, and finally reach consensus. At the early stages, to identify, analyze, and report the themes and subthemes from the interview data, we employed the thematic framework analysis, and developed the preliminary codebooks for data analysis based on the first three transcripts. Then we used continually reviewed transcripts to extend existing themes and identify new ones with a constant comparison method (40, 41).

## Results

This study employed purposive sampling of 75 participants from three towns (X, Y, Z) in Huairou District, Beijing, including 18 village doctors, 15 village officers, 30 residents, 6 town-level community health center managers, and 6 town government managers. The average age of the participants was 57 years, and the majority were female. Tables 1, 2 present participant's basic characteristics.

**Table 1:** Selection of respondents for interviews (%).

**Respondents**	**Town X**	**Town Y**	**Town Z**	**Total**
Village doctors (P1–18)	8 (29.6)	5 (20.8)	5 (20.8)	18 (24.0)
Village officers (P19–33)	5 (18.5)	5 (20.8)	5 (20.8)	15 (20.0)
Residents (P34–63)	10 (37.0)	10 (41.7)	10 (41.7)	30 (40.0)
Town health center managers (P64–69)	2 (7.4)	2 (8.3)	2 (8.3)	6 (8.0)
Town government managers (P70–75)	2 (7.4)	2 (8.3)	2 (8.3)	6 (8.0)
Total	27 (100.0)	24 (100.0)	24 (100.0)	75 (100.0)

* *“P” refers to participant*.

**Table 2:** Basic sociological characteristics of respondents.

**Variables**	**Village doctors**	**Village officers**	**Residents**	**Town health center managers**	**Town government managers**	**Total**
**Age (years)** $\overset{\bar{}}{X}\pm SD$	63.11 ± 11.79	52.93 ± 8.78	60.38 ± 12.40	43.8 ± 7.19	45.83 ± 5.56	57.19 ± 12.35
**Gender**						
Male	10	7	4	2	3	26
Female	8	8	26	4	3	49
**Education**						
Junior high school or lower	12	2	21	0	0	35
High school	3	6	6	0	0	15
College or higher	3	7	3	6	6	25

After in-depth compilation and analysis of the interview data, the role played by village doctors during the pandemic was summarized in three themes as follows: (a) participation in pandemic prevention and control; (b) pandemic prevention in village health offices; and (c) basic medical service delivery. Table 3 shows the themes and sub-themes. Each theme is illustrated with the original words of the participants as examples.

**Table 3:** Themes for village doctors' responsibilities and challenges in the COVID-19 pandemic prevention and control.

**Themes**	**Sub-theme**	**Illustrative quotes**
Participation in pandemic prevention and control	Guiding the village committee to conduct disinfection	“*The village should be disinfected regularly. The village doctor mixed the proportion of disinfectant water, and then the village committee assigned someone to disinfect. Because the village doctor is older, we did not call him (to disinfect).”—P32*
	Monitoring persons in home isolation	“*(Village doctor) monitored the situation of residents in the village at all times and reported any feverish patients to the community health center at once.”—P68*
	Disseminating knowledge on prevention of COVID-19 pandemic	“*Sometimes (village doctor) came over and broadcasted the pandemic prevention knowledge by ‘loudspeaker' in the village.”—P74*
Pandemic prevention measures in village clinics	Distributing village clinic pandemic prevention materials	“*After March 2020, we distributed some pandemic prevention and control materials to village clinics, such as disinfectant alcohol and disinfectant solution, medical masks, etc.”—P64*
	Undertaking village clinic pre-screening	“*When a patient came to the village clinic, he first scanned the ‘health code' and then asked about his epidemiological history. The observation room was set up next door and no (patients in fever) were found. From the COVID-19 outbreak until now, not even patients with fever have been seen, and there are fewer people getting colds.”—P4*
Basic medical service delivery	Common diseases and chronic diseases treatment	“*The village doctor used to study and take exams in the county. The medical skills are fine. Headaches and minor ailments can be diagnosed and treated.”—P39*
	Medicine delivery from the community health center	“*There were a lot of chronic patients in the village. They need to go to the community health center to buy drugs. The village clinic did not have these drugs, and the cost could not be reimbursed. During this pandemic they all could not go out of the village, so the village doctor went to the community health center to help them get medicine.”—P65*
Difficulties and challenges	Lack of pandemic prevention materials	“*During the pandemic, the village doctors had insufficient supplies for pandemic prevention and the budgets for supplies were inadequate. According to the requirements of pandemic prevention norms (wearing protective suit, gloves, masks and face masks and changing them once a day), it actually failed to meet such requirements.”—P71*
	Inadequate capacity of village doctors	“*To be honest, (the village clinic) cannot provide medical treatment; now many diseases cannot be treated, and he will directly tell you to go to the community (health center).”—P57*
	Overage village doctors	“*Village doctors can do few work, and all other work is done by the community health center and village committee staff, because the village doctor team is aging.”—P21*

### Theme 1: Participation in Pandemic Prevention and Control

During the COVID-19 pandemic, rural communities in China adopted social distancing and lockdown measures. Village doctors, as the only health professionals in the village, also took on different levels of pandemic prevention and control tasks. Respondents described what village doctors did during the COVID-19 pandemic from their own perspectives.

### Guiding the Village Committee to Conduct Disinfection

Environmental disinfection is an important measure to kill virus and prevent and control the COVID-19 pandemic. In order to guarantee the residents' health and life safety, the village committee regularly arranged people to carry out environmental disinfection every week. Thus, the village doctor, as a professional, also became part of the sanitization team. Village doctors supervised technicians to do the environmental disinfection in the village, while in other cases technicians personally took the sanitizer devices to disinfect surroundings in the village.

“*I thought the village doctor did play a big role in the pandemic prevention and control. He guided the implementation of various measures, including environmental disinfection. Just now we talked about villager autonomy. Actually there were many villages which did not allow the community health center staff to enter the village. You can only let him (village doctor) do it, as (village doctor) is also a professional. And he has prestige in the village committee, so his words are valid*.” *(P72)*

### Monitoring Persons in Home Isolation

Under confined management, people who are not from the village were not allowed to enter the village. The village clinic is at the bottom of the network of rural three-tier medical services, and in this situation the role of the village doctor is highlighted. The village doctor must not only do his original job, but also take on some of the tasks of the community health center. Medical observation and health monitoring of people in home isolation is an important task. During several outbreaks in China, such as Wuhan pandemic and Beijing Xinfadi pandemic, the government required home quarantine for people who had visited the pandemic outbreak sites. The village doctor was required to record the temperature and symptoms of the home isolates daily and report the data to the community health center.

“*The community health center, the town department in charge of health and the village committee had high requirements on the village doctor, after all the village doctor was a professional. For example, the village doctor identified people in the village who have an abnormal body temperature and reported whether they are returning from outside*.” *(P22)* “*The village doctor followed up with some febrile patients who were seen in secondary and tertiary hospitals, and he may not be a suspicious subject for the COVID-19. But after he returned to the village, someone had to supervise him and take his temperature every day. At this time the community health center may arrange a follow-up visit by a village doctor*.” *(P67)*

### Disseminating Knowledge on Prevention of COVID-19 Pandemic

Based on the information provided by the community health center about the prevention and control of the pandemic, the village doctors disseminated knowledge to villagers, including knowledge on the dynamics of the COVID-19 pandemic, and personal and family protection against infectious diseases.

“*There was no village doctor in our village, but a village doctor from other places drove to the village to educate us on pandemic prevention. There was no broadcast, so the village doctor could only explain to the villagers face to face; there was no village clinic to provide medical services to the villagers, and a company project department was rented as a temporary medical site*.” *(P27)*

In order to improve the ability of village doctors to deal with the COVID-19 pandemic, the community health center regularly conducted training for village doctors.

“*The community health center conducted training for village doctors during the pandemic, on the detection and reporting of suspicious cases, epidemiological investigations, referrals, infection prevention and control, disinfection knowledge and skills, and personal protective measures*.” *(P69)*

### Theme 2: Pandemic Prevention Measures in Village Clinics

When the village was locked down, the village clinic became almost the sole medical facility for the villagers to seek medical treatment. In order to perform the “sentinel” monitoring role of the village clinic, two aspects of work need to be done.

### Distributing Village Clinic Pandemic Prevention Materials

With a variety of patients seeking treatment at the village clinic, it becomes a high-risk site for COVID-19. Therefore, the village clinic has to prepare pandemic prevention and control materials and conduct environmental disinfection to ensure safety in healthcare services to the residents.

“*The community health center handed out masks to us a few times, but the number was small and they told me to use them sparingly, and the alcohol and disinfectant was bought by myself*.” *(P9)* “*(In the village clinic) I had to wear a mask, a white coat, open windows and do ventilation, and disinfect twice a day*.” *(P15)*

### Undertaking Village Clinic Pre-screening

Pre-screening is the means for medical institutions to effectively control infectious diseases and prevent cross-infection within medical institutions. All patients who visited the village clinic must register valid identification information or scan the “health code”. For patients whose diagnosis were not clear and who could not be excluded from infectious diseases, the village clinic should promptly report and take isolation measures for patients.

“*The patient's temperature was taken before he enters the village clinic, and then was asked to scan the ‘health code' and register, and was asked if he had been out of the village and where he had been*.” *(P11)*

### Theme 3: Basic Medical Service Delivery

The village doctor is the gatekeeper of the majority farmers' health. He provides the basic medical services for farmers and alleviates the lack of healthcare and medicine in the vast rural areas of China. The village doctors are usually able to provide timely and brief treatment for common and multiple diseases. In remote rural areas, village doctors play a role that cannot be replaced by urban public hospitals.

### Common Diseases and Chronic Diseases Treatment

Village doctors are the closest medical resource to the villagers. Since the rural elderly population is large and has a greater demand for medical services, village doctors provide initial treatment for common diseases and chronic diseases for villagers. Especially in villages with remote locations and inconvenient traffic, village doctors play an important role as “gatekeepers”.

“*The village doctor is closer to my house, and although expenses of the medicine cannot be reimbursed at the village clinic, it takes a short time to get there by bike. At that time, I ran out of my antihypertensive drugs, so I went to him to buy some*.” *(P39)*

### Medicine Delivery From the Community Health Center

There are many patients with chronic diseases in the village, but provision of the long-term medicine of some patients was affected during the pandemic. To facilitate safe use of drugs and reduce the risk of cross-infection, the village doctor helps patients buy drugs from the community health center and delivers them to their homes.

“*During the pandemic we practiced purchasing on their behalf; we took their health insurance cards and went to the community health center to prescribe drugs for them. We established a procurement team with two people. I was responsible for procurement and the other villager was managing the money. We communicate with the villagers about their demands in WeChat groups*.” *(P18)*

### Theme 4: Difficulties and Challenges

Village doctors have played a great role in pandemic prevention and control, but at the same time, they have also shown shortcomings, which brings great challenges to the construction of rural pandemic prevention and control systems.

### Lack of Pandemic Prevention Materials

At the early stage of the pandemic, due to the lack of a comprehensive emergency supplies reserve system in each region, the shortage of supplies at the grassroots level was prominent. Most health professionals in rural areas only wore daily work clothes and disposable medical masks, which increased the risk of cross-infection through direct physical contact with key populations. Other materials for pandemic prevention and control were also unavailable.

“*The village needs to report and isolate people with fevers, but some rural pandemic prevention materials were inadequate, such as temperature measuring instruments, which are inaccurate sometimes. The tents used at the village entrance checkpoint were not adequate, and these tents do not have a long service lifetime*.” *(P20)*

### Inadequate Capacity of Village Doctors

According to statistical data, more than half of the village doctors have been educated at junior high school level or below. There are even village doctors who are “barefoot doctors” without professional and systematic medical training. The medical knowledge possessed by village doctors is not enough to keep up with medical developments. Outdated knowledge and lack of clinical experience make it difficult for village doctors to identify and report the COVID-19 timely.

“*Because of the limited medical expertise of the village doctor, if he is allowed to practice alone in the village, and a patient infected with COVID-19 failed to report the pandemic history, then everyone is faced with the risk of being infected. The village doctor has an assisting role, responsible for taking temperature, blood pressure and organizing and coordinating. The part that requires professional knowledge will be done by the community health center doctor*.” *(P65)*

### Overage Village Doctors

The results show that the average age of village doctors has exceeded 60 years. Because of low-incomes, high medical risks, and the lack of pension security, it is difficult for rural areas to recruit young village doctors. Difficulties faced by the old village doctors in pandemic prevention and control include being unable to operate with information technology, being easily infected, and having weak awareness.

“*The main difficulty is that the village doctor is too old, and they can't physically withstand the long hours of work. The youngest village doctor is 55 years old now, and the oldest is over 70. So we invite them to work here in the morning and let them rest in the afternoon*.” *(P66)*

## Discussion

This study has explored the roles of and challenges faced by village doctors during the COVID-19 pandemic in rural Beijing. By reporting village doctors' contributions to the prevention and control of COVID-19 in China in the context of worldwide public health emergencies, our study enriches the international discussion of similar topics. The results of interviews showed that village doctors played multiple roles in pandemic prevention and control at the village level, both medical and non-medical. (a) “Sentinel monitors”, which means strictly practicing pre-screening triage system and timely reporting of suspicious patients such as those in fever. (b) “Message examiner”, which means examining ones returning from the medium and high risk areas, and reporting the information to superior departments in time. (c) “Educators”, which means spreading pandemic prevention and control knowledge, policies and medical guidelines to villagers *via* various channels. (d) “Gatekeepers”, which means providing services such as basic medical care and medicine purchase on behalf of patients in the village, delivering medicines to patients at home, as well as making health follow-ups for patients with chronic diseases. (e) “Guidance officer”, which means working as a professional to guide the implementation of health-related work for local people.

Meanwhile, the study found that village doctors are facing multiple challenges in rural COVID-19 pandemic prevention and control efforts, including exposure to serious infection risks due to lack of pandemic prevention materials, the aging village doctor workforce, and inadequate pandemic prevention and control capacity and medical skills. In rural China, rural pandemic prevention and control teams are formed with village officers as leaders and village doctors and volunteers as auxiliaries. The team is tasked with quarantine, health education, and travel restrictions in the village, effectively reducing the spread of the pandemic in rural areas. This is consistent with other studies (42, 43).

Our study found that in the absence of available health care options, village clinics became the sole source of health care services. Village clinics are the tail end of medical service network and also the most broadly covered primary health care institutions (44). The vast majority of village doctors have been practicing for more than 10 years and have established a good doctor-patient relationship with villagers (45, 46). After the pandemic outbreak, village doctors became major forces in the prevention and control of rural pandemics by virtue of their familiarity with villagers. Village doctors do not fear hard work, no matter how much they are paid, or how high the risk is of being infected in the front line of the fight against the pandemic. Some village doctors eventually collapsed in the front line of pandemic prevention and control because of overwork. A number of studies have found that frontline health care workers are under tremendous mental stress and that some are experiencing anxiety and depression, yet without any access to psychological support (47–49). Therefore, health authorities and local governments should pay “pandemic prevention allowance” and humanistic care to village doctors for their contribution and keep them motivated to work.

The research shows that the role of village doctors in the prevention and control of the COVID-19 pandemic in rural areas is mainly evident in both “prevention” and “treatment”. “Prevention” refers to interrupting the spread of COVID-19 pandemic and early detecting suspected cases through pre-screening and triage at early stage, as well as spreading knowledge of COVID-19 prevention and disinfection. Those are also common public health measures taken in China (50, 51). “Treatment” refers to the village doctors' providing medical services and basic public health services to patients with chronic and common diseases in the village. During lockdowns of the village, the community health center medical staff could not enter the village and villagers could not leave. Village doctors undertook basic medical and basic public health services in the village, such as following up with hypertensive and diabetic patients and fetching medicine for villagers.

Severe aging and lack of capacity are critical problems exposed by the village doctor workforce when they are involved in pandemic prevention and control. Many village doctors are transformed from “barefoot doctors” who have received less medical education, resulting in village doctors not meeting the medical needs of residents (12). The data showed that 26.8% of village doctors in China were over 60 years old in 2018, and only 5% were below 35 years old. 93.4% of village doctors are with education in secondary school (high school) or below (52). The proportion of village doctors who aged above 60 in some towns reached 80% (36). The low capacity and overage staff pose challenges for carrying out pandemic prevention and control in three aspects. Firstly, since elderly village doctors practice irregularly, they face higher risk of being infected, and repeated contact with villagers can easily cause cross-infection. Secondly, high-intensity work can cause more physical and psychological stress for village doctors. Thirdly, village doctors do not know how to use information technology, which invariably increases workload and causes low efficiency.

In 2015, six national departments united to issue a document to train free “Tailor-made medical students for rural areas” (53). After graduation, those medical students will be assigned to the town-level community health center or village clinic to practice for a certain number of years. The government hopes to supplement the number of young village doctors and improve the service quality of primary care institutions in this way. In order to achieve the Healthy China and Rural Revitalization Strategy, the Chinese government is formulating policies to strengthen the construction of primary health care service system and talent pool to facilitate the development of village doctors' team. The outbreak of COVID-19 has shown the shortcomings of rural areas in pandemic prevention and control, and also highlighting the role of village doctors. Therefore, it has accelerated the formulation and improvement of related policies from various aspects.

A study has shown a COVID-19 knowledge gap among different populations, with education level and internet media use two mains influential factors (54). This is also true for village doctors and villagers. Village doctors are not informed about the prevention and control protocols and infection characteristics of the COVID-19 in time, which is harmful to early detection of infectious diseases and thus may result in missing of the best period of pandemic prevention and control. Thus, the health department should promptly train village doctors on infectious disease prevention related laws, emergency plans, infection symptoms, etc. A good command of knowledge on the COVID-19 can guarantee that village doctor raises villagers' awareness of pandemic prevention by health education and follow-up visits.

During the pandemic, the rural areas have not yet established comprehensive emergency supply reserve system, and there is an extreme shortage of pandemic prevention and control supplies. Village doctors were in direct and close contact with key populations while only wearing ordinary white coats and disposable masks, which greatly increased the risk of cross-infection. Thus, the government like the Emergency Management Bureau should optimize the medical emergency material supply system so as to promptly equip village doctors with emergency materials. Meanwhile, it is recommended to strengthen the deployment of materials for primary health care institutions. The needs of primary health care institutions should not be ignored in material allocations.

### Strengths and Limitations

Village doctors are an important force in the prevention and control of COVID-19 pandemic in rural China, but the role of and the challenges encountered by village doctors in the prevention and control of COVID-19 pandemic have not been sufficiently and effectively explored. We also provided suggestion for enhancing roles of village doctors in the prevention and control of COVID-19 pandemic in rural areas in order to improve the public health emergency system in rural China. Meanwhile, the experiences from China's rural areas in pandemic prevention and control can provide a reference for rural areas in other countries. This study has several limitations. First, we recruited interviewers from only one district in Beijing, and the responsibilities of village doctors may vary among districts. Second, the pandemic was already under control when we interviewed, so the results may have recall bias. Third, because of the strict pandemic prevention and control in some pandemic outbreak areas, we were not able to interview the relevant personnel to fully understand the role of village doctors in the pandemic outbreak areas.

## Conclusions

We interviewed five groups of participants in Huairou District, Beijing, to understand the contribution of village doctors when the strict COVID-19 quarantine measures were implemented, and analyzed the difficulties and challenges they have faced in pandemic prevention and control. Village doctors played the role of educators, messengers, monitors, gatekeepers, and instructors in pandemic prevention and control. Their work included guiding the disinfection, providing basic health care, monitoring the temperature of people isolated at home, screening the information of people returning to the village, and educating on the prevention of infectious diseases. They do their best to use their expertise to contain the spread of COVID-19 in rural areas. But the fragile public health system in rural areas is highly vulnerable to public health emergencies. Lack of rural pandemic prevention materials, low medical level of village doctors and aging staff structure have also become the weaknesses of rural pandemic prevention and control. The outbreak of the COVID-19 pandemic should prompt the government to invest in more skilled manpower, material and financial resources in the rural health care system, especially in village clinics and village doctors. In preparation for any public health emergencies at any time, great efforts should be made to upgrade medical equipment in village clinics and to improve pension security and salaries for village doctors to attract more young talents to work as village doctors.

## Data Availability Statement

The raw data supporting the conclusions of this article will be made available by the authors, without undue reservation.

## Ethics Statement

The studies involving human participants were reviewed and approved by the Ethics Committee of Capital Medical University (Z2022SY021). The participants signed an informed consent form for their participation in this study. The patients/participants provided their written informed consent to participate in this study.

## Author Contributions

JL and JY conceived and designed of the study, and contributed to the revision of the manuscript. NZ, HY, and HZ collated the database. NZ and JL performed the data analysis. JL wrote the first draft of the manuscript. All authors contributed to the interview data collection. All authors contributed to reviewing and proofreading the manuscript and approved the submitted version.

## Funding

This work was supported by the Capital Health Management and Policy Research Base, China (2021JD06), and the Beijing Social Science Foundation (20JCB004), and the Beijing Education Commission (SZ201910025008).

## Conflict of Interest

The authors declare that the research was conducted in the absence of any commercial or financial relationships that could be construed as a potential conflict of interest.

## Publisher's Note

All claims expressed in this article are solely those of the authors and do not necessarily represent those of their affiliated organizations, or those of the publisher, the editors and the reviewers. Any product that may be evaluated in this article, or claim that may be made by its manufacturer, is not guaranteed or endorsed by the publisher.
