# Supplementary material for: Roles and Challenges for Village Doctors in COVID-19 Pandemic Prevention and Control in Rural Beijing, China: A Qualitative Study
Source: Front Public Health. 2022 Jun 29;10:888374. doi: 10.3389/fpubh.2022.888374 (PMC9277090; doi:10.3389/fpubh.2022.888374)
Supplement: Supplementary file 1 [file Data_Sheet_1.PDF]

Additional Material

Interview outline (Village doctors)

1. Basic Information of village doctors

(1) Gender______

①Male ②Female

(2) Age_______(years)

(3) Household Registration______

①This village ②Other villages in the township ③Other townships in Huairou District ④Other

(4) The monthly income of being village doctors______(yuan)

(5) Academic qualifications______

①Elementary school and below ②Junior High School ③High School ④Secondary College ⑤Junior college ⑥Bachelor and above

(6) Your current qualifications______

①None ②Certificate of Village Doctor ③Certificate of Rural General Practitioner (assistant) ④Certificate of Assistant Medical Practitioner ⑤ Certificate of Medical Practitioner

(7) When did you start your career as a village doctor (barefoot doctor)?______(year)

2. Basic information of village clinics

(1) Where is your village clinic room from?______

①Free use (provided by_______) ②Own house ③Rent, the rent is _____Yuan/year

(2) How many square meters is the area of the village clinic?______

(3) What is the layout of the village clinic building?

①four separate rooms ②three separate rooms ③two separate rooms ④only one room

(4) Does the village clinic have a separate observation room? _____ What is the approximate size of the room?______

(5) Does your village clinic receive government purchased services?

3. Epidemic prevention and control situation

(1) Do you know the 10 need-to-know for village clinic epidemic prevention and control issued by the National Health Commission?

(2) In the past year, what epidemic prevention and control efforts have you participated in in your village?

(3) What were the varieties of prevention and control materials that village doctors had during the epidemic level 1 response? Was it sufficient?

(4) What were the varieties of prevention and control materials that village doctors had during the epidemic prevention and control normalization? Was it sufficient?

(5) How did you carry out personal protection during the epidemic?

(6) Did you treat febrile patients during the epidemic? What did you do if you received a febrile patient?

(7) What are the differences in the residents who came to the village clinics during the epidemic compared to those before the epidemic?（Hint: number of people, type of patients)

(8) Can the village clinic meet the daily medical service needs of the villagers during the epidemic? How did patients get the medical services and medicines they needed?

(9) Combining the epidemic prevention and control work in your village, what do you think are the difficulties and challenges in epidemic prevention and control work in rural areas?

(10) From a long-term perspective, what should be done to strengthen the epidemic prevention, control and handling capacity of village doctors?

Interview outline (Village officers)

1. Basic Information of village officers

(1) Gender______

①Male ②Female

(2) Age_______(years)

(3) Academic qualifications______

①Elementary school and below ②Junior High School ③High School

④Secondary College ⑤Junior college ⑥Bachelor and above

(4) What year did you start serving on the village committees?______ What is your current position on the village committees?______

(5) How much did you earn per month as a village officer last year?______(yuan)

2. Village clinic and health service utilization

(1) Are the medical services currently provided by village doctors able to meet the villagers' medical needs?

(2) Will the village clinic be able to achieve the goal of " minor illnesses not leaving the village "? What are the difficulties and areas for improvement in the village clinics?

(3) What do you think are the shortcomings of village doctors in this village?

(4) What knowledge and skills do you think village doctors should have?

3. Epidemic Prevention and Control

(1) The registered population of your administrative village are ________ and the resident population are ________. The area of the village is approximately ________ square kilometers.

(2) What were the village committees responsible for the prevention and control of the

epidemic in the village?

(3) Which work of village doctors is managed by village committees in epidemic prevention and control?

(4) What kind of epidemic prevention and control work does the village doctor undertake in the village?

(5) Combining the epidemic prevention and control work in your village, what do you think are the difficulties and challenges of the epidemic prevention and control work of village doctors?

(6) How was the role played by the village doctor in your village in the epidemic prevention and control work?

(7) From a long-term perspective, which aspects should strengthen the village doctors' ability to prevent and control epidemic?

Interview outline (Residents)

1. Basic Information of residents

(1) Gender______

①Male ②Female

(2) Age_______(years)

(3) Academic qualifications______

①Elementary school and below ②Junior High School ③High School

④Secondary College ⑤Junior college ⑥Bachelor and above

(4) How much did your family earn in total last year?______(yuan)

(5) Do you have any chronic diseases?

2. Village clinic and health service utilization

(1) Will the village clinic be able to achieve the goal of " minor illnesses not leaving the village "?

(2) Are you worried about access to medical care in the village in the future?

(3) What do you think are the shortcomings of village doctors in this village?

(4) What knowledge and skills do you think village doctors should have?

(5) Have you visited the village clinic in the past year? (If you choose yes, please continue to answer) Can you tell us about your experience at the time of your visit? Were you satisfied with your visit?

(6) Do you think the basic medical services currently provided by the village clinic can meet your health needs?

(7) What are the main problems facing the work of basic medical and public health

services provided by village clinics?

3. Epidemic Prevention and Control

(1) How did you protect yourself during the epidemic?

(2) Did you have trouble getting medical care and buying medicine during the epidemic? How were these difficulties resolved?

(3) Did the village doctor provide health care services to meet your medical needs during the epidemic?

(4) Are you know the work of village doctors in the prevention and control of the epidemic? (If yes, please continue) What tasks were village doctors involved in the prevention and control of epidemics in the village?

(5) What aspects of the capacity of village doctors do you think need to be strengthened during the epidemic prevention and control?

Interview outline (Township healthcare center managers)

1. Basic Information of township healthcare center managers

(1) Gender______

①Male ②Female

(2) Age_______(years)

(3) Academic qualifications______

①Junior High School ②High School/Secondary College ③Junior college

④Bachelor ⑤Master ⑥Other

(4) What year did you work at this township healthcare center and what year did you become the manager?

(5) Position______

①Director/Dean ②Vice Director /Vice Dean

(6) Professional and technical titles______

①Staff Grade ②Elementary ③Intermediate ④Deputy Chief Physician ⑤Chief Physician

(7) What is your annual income?_____(yuan)

2. Medical Service Delivery

(1) What basic public health services can village doctors provide?

No.	Basic public health service programs	Provide contents	Appraisal indicators
1	Health record management		
2	Health education		
3	Preventive vaccination		
4	Health management for children aged 0-6 years		
5	Maternal health management		
6	Elderly health management		

7	Health management of hypertensive patients		
8	Health management of type 2 diabetic patients		
9	Management of seriously mentally patients		
10	Reporting and handling of infectious diseases and public health emergencies		
11	Health supervision and assistant management services		

(2) Do you think the public health services provided by village doctors can meet the needs of rural residents? What aspects need to be improved or strengthened?

(3) What basic medical services can village doctors provide?

No.	Basic medical service programs	Provide contents	Appraisal indicators
1	General common and multiple disease treatment		
2	Emergency rescue		
3	Family medical services		
4	Referral services		
5	Rehabilitation medical services		
6	Other medical services		

(4) Do you think the basic medical services provided by village doctors can meet the

needs of rural residents? What aspects need to be improved or strengthened?

3. Epidemic Prevention and Control

(1) What services did the village doctors provide during the epidemic?

(2) What were the channels for village doctors to obtain epidemic prevention materials?

(3) What were the varieties of prevention and control materials that village doctors had during the epidemic level 1 response? Was it sufficient?

(4) What were the varieties of prevention and control materials that village doctors had during the epidemic prevention and control normalization? Was it sufficient?

(5) What aspects of epidemic prevention and control knowledge training were provided to village doctors during the epidemic by the township healthcare centers? How did you think the training was effective?

(6) Can the village clinic meet the daily medical service needs of the villagers during the epidemic? How did patients get the medical services and medicines they needed?

(7) What role did you think village doctors played during the epidemic prevention and control?

(8) what do you think are the difficulties and challenges in epidemic prevention and control work in rural areas?

(9) From a long-term perspective, what should be done to strengthen the epidemic prevention, control and handling capacity of village doctors?

Interview outline (Township government managers)

1. Basic Information of township government managers

(1) Gender______

①Male ②Female

(2) Age_______(years)

(3) Academic qualifications______

①High School ②Junior college ③Bachelor ④Master

(4) What year did you start serving in township government?______(year)

(5) Your current township Position is _________

①Secretary ②Township Manager ③Vice Secretary ④Vice Township Manager

⑤Township Public Health Management Committee Member ⑥Other

2. Townships governments and epidemic prevention and control

(1) Your township has ______ administrative villages, a town area of ______ square kilometers, a registered population of ______ and a usual population of ______.

(2) Which department in the township government is currently primarily responsible for public health? What are the main tasks?

(3) Has the township government developed specific documents for the prevention and control of the epidemic in the township? What was the main content around which the document was developed?

(4) Has the township government set up an epidemic prevention and control workgroup? (If yes, continue asking) What is the composition of the prevention and control group? What are the responsibilities of the division of labor?

(5) What were the main aspects of epidemic prevention and control and public health management by the township government to the village committee?

3. Village doctors and epidemic prevention and control

(1) What kind of epidemic prevention and control work did the village doctors undertake in the village during the epidemic prevention and control?

(2) As far as you know, what are the main problems facing the village doctor workforce in this township at present?

(3) What do you think were the difficulties and challenges for village doctors when they were involved in the prevention and control of epidemic?

(4) In the epidemic prevention and control, did the township government have special funds or materials for village doctors?

(5) From a long-term perspective, what should be done to strengthen the epidemic prevention, control and handling capacity of village doctors?

(6) What do you think should be done to establish a long-term mechanism for the prevention and control of epidemic in rural areas?
